# Supplementary material for: Deep-embedded clustering by relevant scales and genome-wide association study in autism
Source: PLoS One. 2025 May 29;20(5):e0322698. doi: 10.1371/journal.pone.0322698 (PMC12122041; doi:10.1371/journal.pone.0322698)
Supplement: Attachment — Submitted filename: Response to Reviewers.docx [file pone.0322698.s002.docx]

**Point-by-point response to reviewers and editors**

**Response to Journal Requirements**

**1. Please ensure that your manuscript meets PLOS ONE's style requirements, including those for file naming.**

We have checked that our submission, including the file name, meets PLOS ONE's style.

**2. Please provide an amended statement that declares *all* the funding or sources of support (whether external or internal to your organization) received during this study, as detailed online in our guide for authors at http://journals.plos.org/plosone/s/submit-now. Please also include the statement “There was no additional external funding received for this study.” in your updated Funding Statement. Please include your amended Funding Statement within your cover letter. We will change the online submission form on your behalf.**

Thank you for pointing this out.

We have added and modified the following text in both the online form and cover letter.

*The present study was supported by the Ministry of Education, Culture, Sports, Science and Technology (MEXT) KAKENHI Grant Numbers 19H03894, 21K17304 and 22H03346. The funders had no role in study design, data collection and analysis, decision to publish, or preparation of the manuscript. There was no additional external funding received for this study.*

**3. All PLOS journals now require all data underlying the findings described in their manuscript to be freely available to other researchers, either a. In a public repository, b. Within the manuscript itself, or c. Uploaded as supplementary information. This policy applies to all data except where public deposition would breach compliance with the protocol approved by your research ethics board. If your data cannot be made publicly available for ethical or legal reasons (e.g., public availability would compromise patient privacy), please explain your reasons on resubmission and your exemption request will be escalated for approval.**

Thank you for sharing the journal's policy regarding data sharing. We recognize that making data publicly available is very important to ensure highly replicable research. However, the datasets analyzed in this study are subject to the Simons Foundation's policy and cannot be made publicly available. However, the dataset analyzed in this study is available upon request from the corresponding author. The following content has been added to the **"Data and materials availability**” section.

***Page 28, Lines 378–384***

***Data and materials availability***

*The data used in this study are available only to those who are granted access by the Simons Foundation due to restrictions related to participant privacy and ethical considerations. The Simons Foundation’s data access policy ensures that sensitive genetic and phenotypic data of participants are protected in accordance with ethical guidelines and data protection regulations. Researchers who wish to access the data must submit a request to the SFARI Data and Biospecimen Repository (SDBR). Data access requests can be directed to the SDBR at [sdbr@simonsfoundation.org].*

**Response to comments**

**Reviewer 1**

1) **How did the authors define “ethnicity” in the cluster analysis? Was it based on genetic data or self-reported information?**

The information on the ethnicity used for clustering was obtained from the demographics information in the SPARK database. This is information that was self-reported by the caregivers. We have added information about this to the manuscript.

***Page 5, Lines 104–109***

*We conducted cluster analyses using the phenotypic variables of DCDQ (15 items),SCQ (40 items), and RBS-R (43 items) scoring; age at initial registration in months; self-reported* *ethnicity; dominant hand; and history of medication, biomedical intervention (e.g., diet, alternative medicine, and supplements), and intensive behavioral intervention (e.g., Applied Behavior Analysis, Verbal Behavior, Pivotal Response Treatment). Missing data were imputed using the mean value of each variable, and all categorical data were transformed into dummy variables.*

2) **It is unclear what covariates were used in the GWAS analysis. At a minimum, covariates such as sex, age, and genetic principal components (PCs) should be included. This omission may increase the risk of false positives in the cGWAS results.**

Thank you for your valuable comment regarding the inclusion of children’s sex, age, and principal components (PCs) as covariates in the GWAS analysis.

Regarding sex, because the present study focuses solely on male children (Page 5, Lines 87–88), correction for sex was not deemed necessary.

Age was not included as a covariate in the present GWASs because it was used as a variable for clustering (Page 5, Line 105).

Herein, PC analysis was performed on the genotype data to excluded 33 individuals whose data points exceeded six standard deviations for PCs 1 or 2 (Page 5, Lines 95–101). As the exclusion of these outliers effectively removed participants with substantial ancestry-related variability in the genetic architecture, we concluded that it was unnecessary to include PCs as additional covariates in the GWAS model.

We hope this explanation provides clarity regarding our approach and reassures you that sex, age, and PCs were appropriately addressed, ensuring the robustness of our findings. These explanations have been added to the manuscript.

***Pages 7–8, Lines 155–161***

*For GWAS, no covariates were adjusted for in this study. Owing to the focus of the present study on males, the sex of children was not adjusted for, whereas age was not included as a covariate in the GWAS because it was used as a variable for clustering. Additionally, variables related to genetic architecture were not corrected for in the GWAS because populations with considerable genetic heterogeneity were excluded based on the SNP array data analysis via principal component analysis.*

3) **Did the authors assess linkage disequilibrium (LD) between the 90 identified loci? I suspect that several loci may be in strong LD. If so, please report only the independent signals. A conditional GWAS analysis could also help verify this.**

Thank you for your very valuable comments. LD clumping is important for improving the interpretability of the results. Based on the comments, we performed LD clumping on the GWAS results and identified 90 loci were that were reduced to 27. We have made the necessary additions and corrections to the manuscript and Table 2 accordingly.

***Page 8, Lines 161–162***

*The GWAS results obtained were clumped using linkage disequilibrium, and the locus with the lowest p-value was selected.*

***Page 2, Line 34***

27 chromosomal loci

***Page 12, Line 188***

27 chromosomal loci

**Table 2. Association table of the cluster-based genome-wide association study.**

| **Cluster no.** | **Chr** | **hg19** | **REF** | **ALT** | **ALT_FREQ** | **OR** | **95% CI** | ***p-*value** | **Function** | **Gene symbol** |
| --- | --- | --- | --- | --- | --- | --- | --- | --- | --- | --- |
| 5 | 8 | 77348203 | C | T | 33.67% | 2.61 | (1.86–3.66) | 2.60 × 10^8^ | ncRNA_intronic | LINC01111 |
| 7 | 7 | 13304197 | C | G | 1.90% | 15.46 | (5.94–40.23) | 2.00 × 10^8^ | intergenic | ARL4A, ETV1 |
| 8 | 6 | 169690571 | A | G | 2.06% | 12.81 | (5.18–31.68) | 3.34 × 10^8^ | intergenic | THBS2, WDR27 |
| 8 | 9 | 107163339 | G | A | 1.57% | 13.51 | (5.33–34.25) | 4.10 × 10^8^ | intergenic | LOC105376194, OR13F1 |
| 9 | 12 | 125253297 | C | G | 1.10% | 59.17 | (13.86–252.6) | 3.59 × 10^8^ | intergenic | NCOR2, SCARB1 |
| 14 | 14 | 83919384 | A | C | 1.76% | 13.53 | (5.31–34.44) | 4.72 × 10^8^ | intergenic | LINC02301, SNORD3P3 |
| 16 | 9 | 3529465 | C | G | 2.67% | 8.53 | (4.05–17.96) | 1.71 × 10^8^ | intergenic | RFX3, RFX3-AS1 |
| 17 | 7 | 7702162 | C | G | 2.30% | 7.43 | (3.64–15.18) | 3.68 × 10^8^ | intronic | RPA3, UMAD1 |
| 19 | 5 | 44729840 | C | T | 1.84% | 38.83 | (10.91–138.2) | 1.61 × 10^8^ | intergenic | LINC02224, MRPS30-DT |
| 19 | 5 | 45046992 | T | C | 1.70% | 49.39 | (12.73–191.7) | 1.74 × 10^8^ | intergenic | MRPS30, HCN1 |
| 19 | 8 | 27064581 | A | G | 1.01% | 58.42 | (13.95–244.64) | 2.59 × 10^8^ | intergenic | MIR548H4, STMN4 |
| 21 | 10 | 127919286 | G | A | 1.03% | 49.87 | (13.06–190.4) | 1.07 × 10^8^ | intronic | ADAM12 |
| 23 | 15 | 67441517 | C | T | 1.11% | 84.58 | (22.43–318.94) | 5.64 × 10^11^ | intronic | SMAD3 |
| 23 | 22 | 48720236 | G | T | 1.09% | 54.59 | (12.97–229.69) | 4.88 × 10^8^ | intergenic | MIR3201, TAFA5 |
| 24 | 8 | 2803219 | C | A | 1.84% | 46.49 | (12.26–176.22) | 1.64 × 10^8^ | intronic | CSMD1 |
| 24 | 9 | 116350142 | G | A | 1.34% | 46.83 | (12.35–177.52) | 1.54 × 10^8^ | intronic | RGS3 |
| 24 | 17 | 15166354 | C | T | 1.75% | 44.82 | (11.57–173.67) | 3.74 × 10^8^ | intronic | PMP22 |
| 25 | 1 | 42241824 | C | T | 2.01% | 10.99 | (4.66–25.92) | 4.40 × 10^8^ | intronic | HIVEP3 |
| 25 | 14 | 32339580 | G | A | 1.49% | 13.82 | (5.49–34.79) | 2.52 × 10^8^ | intergenic | NUBPL, LINC02313 |
| 28 | 1 | 186735538 | G | A | 1.04% | 30.26 | (9.01–101.57) | 3.42 × 10^8^ | intergenic | PACERR, PLA2G4A |
| 28 | 3 | 155821228 | A | C | 1.15% | 40.14 | (11.62–138.63) | 5.26 × 10^9^ | intergenic | GMPS, KCNAB1 |
| 31 | 7 | 147512932 | G | T | 1.96% | 32.64 | (9.64–110.51) | 2.13 × 10^8^ | intronic | CNTNAP2 |
| 31 | 17 | 35386339 | T | C | 1.78% | 44.07 | (11.57–167.91) | 2.91 × 10^8^ | intronic | AATF |
| 35 | 20 | 57142361 | G | T | 1.09% | 18.43 | (6.52–52.11) | 3.91 × 10^8^ | ncRNA_intronic | APCDD1L-DT |
| 37 | 19 | 20047738 | T | G | 6.34% | 7.63 | (3.68–15.82) | 4.57 × 10^8^ | intergenic | ZNF93, ZNF682 |
| 39 | 17 | 27641326 | G | A | 1.15% | 20.51 | (6.94–60.62) | 4.69 × 10^8^ | intergenic | NUFIP2, MIR4523 |
| 39 | 17 | 63634041 | G | A | 1.14% | 25.40 | (8.02–80.46) | 3.84 × 10^8^ | intronic | CEP112 |

REF, reference allele; ALT, alternative allele; ALT_FREQ, alternative allele frequency; OR: odds ratio; CI, confidence interval.
