# Supplementary material for: Deep-embedded clustering by relevant scales and genome-wide association study in autism
Source: PLoS One. 2025 May 29;20(5):e0322698. doi: 10.1371/journal.pone.0322698 (PMC12122041; doi:10.1371/journal.pone.0322698)
Supplement: Attachment — Submitted filename: Response to Reviewer Comments.docx [file pone.0322698.s001.docx]

**Comment**
**We understand that you obtained ethical approval from your local ethics committee after you started recruiting participants. PLOS ONE does not accept retrospective ethical approval for human subjects research. As a result, we cannot consider the manuscript for publication at PLOS ONE. We recognize that you obtained ethical approval from your local ethics committee after you began the study: your ethics approval document is dated Dec 21 2020, while personal data were collected and accessed beginning in 12/03/2019. For human subjects research, PLOS ONE requires that authors obtain ethics committee approval before beginning the study, and we do not accept retrospective ethical approval.**

**Response**
Thank you very much for your detailed review and feedback regarding our manuscript submission. We understand and respect the ethical standards upheld by PLOS ONE and acknowledge the concerns raised about retrospective ethical approval. We wish to clarify that the data were initially obtained on December 3, 2019, for a different study (<https://www.nature.com/articles/s41398-020-00951-x>) that had already received ethical approval. The analysis for the current study began on October 1, 2021, with the necessary ethical approvals in place. Therefore, the study is not retrospective in nature. We apologize for any confusion caused by our initial submission and have modified the methods section accordingly.

*Methods (Page 4, Line 71-77)*
This study adhered to the guidelines of the Declaration of Helsinki [15] and all other relevant guidelines. The Institutional Review Board of Tohoku University Graduate School of Medicine (2020-1-826) approved our protocol, and written informed consent was obtained from participants through the Simons Foundation Autism Research Initiative (SFARI) for the SPARK study, which began recruitment on 21/04/2016, and is ongoing. [12]. The data were initially obtained on 03/12/2019, for a different study and were accessed for research purposes at 01/10/2021.
